# Reversing PD-1 Resistance in B16F10 Cells and Recovering Tumour Immunity Using a COX2 Inhibitor

**DOI:** 10.3390/cancers14174134

**Published:** 2022-08-26

**Authors:** Chenyu Pi, Ping Jing, Bingyu Li, Yan Feng, Lijun Xu, Kun Xie, Tao Huang, Xiaoqing Xu, Hua Gu, Jianmin Fang

**Affiliations:** 1 School of Life Sciences and Technology, Tongji University, Shanghai 200092, China; 2 College of Medicine, Henan University of Science and Technology, Luoyang 471000, China; 3 Biomedical Research Center, Suzhou 230031, China; 4 Shanghai Tongji Hospital, Shanghai 200065, China

**Keywords:** programmed death-ligand 1, cyclooxygenase-2, tumour resistance, immunosuppression

## Abstract

**Simple Summary:**

Some patients develop drug resistance to programmed cell death protein 1/programmed death-ligand 1(PD-1/PD-L1) therapy but the mechanism is unclear. Therefore, the study of drug resistance to PD-1 therapy is quite important. In this sense, we obtained B16F10-R tumours resistant to anti-PD-1 therapy through multiple rounds of drug resistance screening in vitro. We found that COX2 expression was significantly elevated and COX2 inhibitors in combination with anti-PD-1 monoclonal antibodies (mAbs) could reverse this resistance phenomenon. Knockout of the ptgs2 gene in B16F10-R tumours also restored tumour sensitivity to anti-PD-1 therapy. Therefore, we believe that the combination of COX2 inhibitors and anti-PD-1 mAbs may become a new choice for the drug resistance of anti-PD-1 therapy in the future.

**Abstract:**

Immunotherapy is an effective method for tumour treatment. Anti-programmed cell death protein 1 (PD-1) and anti-programmed death-ligand 1 (PD-L1) monoclonal antibodies play a significant role in immunotherapy of most tumours; however, some patients develop drug resistance to PD-1/PD-L1 therapy. Cyclooxygenase-2 (COX2) is expressed in various solid tumours, and prostaglandin E2 (PGE2) drives the development of malignant tumours. We developed a drug-resistant B16F10 (B16F10-R) tumour mouse model through four rounds of selection in vivo. Subsequently, we investigated changes in PD-L1 expression and lymphocyte infiltration in B16F10-NR and B16F10-R tumours. Additionally, we explored the role of COX2 in acquired resistance to pembrolizumab, an anti-PD-1 treatment. Immune cell infiltration was significantly decreased in resistant tumours compared to B16F10-NR tumours; however, ptgs2 gene expression was significantly elevated in resistant tumours. Aspirin or celecoxib combined with pembrolizumab can effectively reverse tumour drug resistance. In addition, ptgs2 knockout or the use of the EP4 inhibitor E7046 abrogated drug resistance to anti-PD-1 treatment in B16F10-R tumour cells. Our study showed that inhibition of the COX2/PGE2/EP4 axis could increase the number of immune cells infiltrating the tumour microenvironment and recover drug-resistant tumour sensitivity to pembrolizumab. Thus, we highlight COX2 inhibition as a promising therapeutic target for drug-resistant tumours for future consideration.

## 1. Background

In recent years, tumour immunotherapy has made major strides in cancer treatment [1]. The PD-1/PD-L1 pathway plays an important role in the immunosuppressive meshwork [2,3]. PD-1 is highly expressed on T cells and natural killer (NK) cells, whereas PD-L1/PD-L2 is expressed on antigen-presenting cells and various solid tumour cells. PD-1/PD-L1 interactions suppress T-cell immunity, leading to T-cell exhaustion, anergy, or apoptosis [4,5]. Anti-PD-1 treatment improves antitumour immune responses in patients with colorectal cancer, melanoma, renal cell carcinoma, non-small cell lung cancer, haematologic malignancies, and bladder cancer, resulting from its ability to transform anergic T cells into functional T cells [6,7,8]. However, the objective response rate of anti-PD-1/PD-L1 antibody therapy is approximately 10–20% for most malignancies [9,10]. Studies have shown that the tumour tissue of some tumour patients has congenital insensitivity or resistance to anti-PD-1 treatment [11]. The initial treatment showed a positive effect, but drug resistance was soon acquired in some patients [12]. However, the mechanism for primary and adaptive resistance to anti-PD-1 therapy was unclear.

Anti-PD-1 therapy can increase the expression of inflammatory cytokines, which may counteract its therapeutic effects [13,14]. Cyclooxygenase (COX) is an important rate-limiting enzyme for the conversion of arachidonic acid into various prostaglandins in the body, and it can be divided into at least two subtypes: COX1 and COX2. Unlike COX1, which is present in most tissues, COX2 expression is induced by cytokines and growth factors, and increases rapidly in response to inflammatory stimuli. COX2 activation produces prostaglandin E2 (PGE2), which is associated with enhanced tumour cell survival, migration, growth, angiogenesis, invasion, and immunosuppression [15]. COX2 is expressed in a variety of solid tumours such as colorectal cancer, nasopharyngeal carcinoma, gastrointestinal malignancies, and breast cancer [16,17,18,19]. In addition, it has been demonstrated that PGE2 could drive the development of malignant tumours [20,21,22,23,24,25]. PGE2 is significantly conserved in human cutaneous melanoma biopsies, and it is required for mutant BrafV600E mouse melanoma cell growth [20]. Inhibiting COX2 and PGE2 in colon cancer models enhanced anti-vascular endothelial growth factor therapy and suppressed angiogenesis and tumour growth [25]. In addition, blocking COX2/PGE2-mediated wound response could abrogate bladder cancer chemoresistance [22].

This study investigated the function of COX2 in anti-PD-1 acquired resistance by developing a drug-resistant B16F10 tumour mouse model. Our study showed that COX2 derived from tumours plays an essential role in adaptive tumour resistance. Inhibiting the COX2/PGE2/EP4 axis could increase the number of infiltrating T and NK cells in the tumour microenvironment (TME) and recover drug-resistant tumour sensitivity to pembrolizumab.

## 2. Materials and Methods

### 2.1. Mice and Cells

Human *Pdcd1* transgenic mice were housed in a pathogen-free environment at the Laboratory Animal Research Centre, Tongji University, as previously described [26]. All animal experiments were performed in accordance with the Animal Ethics Committee of Tongji University.

B16F10 melanoma cells were purchased from the American Type Culture Collection (Rockville, MD, USA). B16F10-NR and B16F10-R melanoma cell lines were generated by four rounds of selection for anti-PD-1 resistance. B16F10-R-knockout (KO) *ptgs2* melanoma cells were generated using the CRISPR-Cas9 system (Cas9-2hitKO). The guide RNA sequences targeting *ptgs2* were sgRNA-1, 5′-GCTTTACAGACTTAAAAGCA-3′ and sgRNA-2, 5′-TTCAAGACAGATCATAAGCG-3′. All cells were maintained in Dulbecco’s modified Eagle’s medium (DMEM; Hyclone, Waltham, MA, USA) supplemented with 1% penicillin/streptomycin (Gibco, Waltham, MA, USA) and 10% foetal bovine serum (Gibco) at 37 °C and 5% CO_2_.

### 2.2. Animal Experiments

B16F10, B16F10-NR, B16F10-R, and B16F10-R-knockout *ptgs2* cells (1 × 10^5^ cells) were resuspended in phosphate-buffered saline PBS (Cytiva, Marlborough, MA, USA) and injected subcutaneously into the flanks of 6–8-week-old female human *Pdcd1* transgenic mice. Mice were randomised into groups, each comprising 6–8 mice. When tumours grew to 50 mm^3^, mice were administered pembrolizumab (10 mg/kg; MSD, USA) or the control isotype for ophthalmic intravenous injection twice a week, and aspirin (10 mg/kg; Selleck, Shanghai, China), SC560 (5 mg/kg; Selleck), celecoxib (5 mg/kg; Selleck), and E7046 (10 mg/kg; Selleck) for intraperitoneal injection three times a week. The longest dimension (length) and longest perpendicular dimension (width) were measured every two days using a calliper. Tumour volume (mm^3^) = (length × width × width)/2. For in vivo B16F10-R cell selection, B16F10 tumours were digested with trypsin and collagenase until reaching 1500 mm^3^, which was the humane endpoint. CO_2_ inhalation was used to euthanise the mice. The cells were resuspended in DMEM and cultured for two weeks. The tumour cells were re-injected subcutaneously into another mouse for subsequent rounds of in vivo selection.

### 2.3. Real-Time Quantitative Polymerase Chain Reaction (RT-qPCR)

Total RNA was extracted using the Animal Tissue/Cell Total RNA Extraction Kit (DAKEWE, Beijing, China) and cDNA was acquired using a reverse transcription kit (Solarbio, Beijing, China). *Ptgs1/ptgs2* transcript levels were measured using an RT-PCR SYBR Green I kit (Solarbio). RT-qPCR was performed on a ROCHE LightCycler^®^ 96 (Roche, Basel, Switzerland) in a 96-well plate, and each sample was prepared in triplicates. The following primer pairs were used: GAPDH, forward primer 5′-TGGCCTTCCGTGTTCCTAC-3′ and reverse primer 5′-GAGTTGCTGTTGAAGTCGCA-3′; mouse COX1, forward primer 5′-TTACTATCCGTGCCAGAACCA-3′ and reverse primer 5′-CCCGTGCGAGTACAATCACA-3′; mouse COX2, forward primer 5′-AGCAAATCCTTGCTGTTCCAA-3′ and reverse primer 5′-GCAGTAATTTGATTCTTGTC-3′. 

### 2.4. Flow Cytometry

Tumours were cut and digested with trypsin and collagenase. A 40 µm mesh filter (BioFIL, Shanghai, China) was used to filter the single-cell suspension (BioFIL, Shanghai, China). The cells were blocked with purified rat anti-mouse CD16/CD32 (553142; BD Pharmingen, San Diego, CA, USA), and dead cells were stained with BD Horizon Fixable Viability Stain 510 (564406; BD Pharmingen). Cell surface staining was performed using the following antibodies: APC-Cy™7 mouse anti-mouse CD45.2 (560694; BD Pharmingen), PE hamster anti-mouse CD3e (553063; BD Pharmingen), PerCP-Cy™5.5 rat anti-mouse CD4 (550954; BD Pharmingen), FITC rat anti-mouse CD8a (553030; BD Pharmingen), APC anti-mouse NK-1.1 antibody (108710; BioLegend, San Diego, CA, USA), FITC anti-mouse CD49b (pan-NK cells) antibody (108905; BioLegend), PE/Cyanine7 anti-mouse TCR β chain antibody (109222; BioLegend), and PE anti-mouse CD274 (B7-H1, PD-L1) antibody (124308; BioLegend). The following antibodies were used as isotype controls: APC-Cy™7 Mouse IgG2a, κ Isotype Control (557751; BD Pharmingen), PE Hamster IgG1 κ Isotype Control (553972; BD Pharmingen), PerCP-Cy™5.5 Rat IgG2a, κ Isotype Control (550765; BD Pharmingen), FITC Rat IgG2a, κ Isotype Control (553929; BD Pharmingen), APC Mouse IgG2a, κ Isotype Control (551414; BD Pharmingen), FITC Rat IgM, κ Isotype Control (555951; BD Pharmingen), PE Rat IgG2b, κ Isotype Control (553989; BD Pharmingen). All analyses were performed using a CytoFLEX LX (Beckman Coulter, Brea, CA, USA).

### 2.5. PGE2 Concentration Detection

Cells were plated at 0.5–1 × 10^6^ cells/mL in 96-well plates at 37 °C in the absence or presence of 100 mL of conditioned medium from tumour cells plus or minus LPS (10 to 100 ng/mL) in a total volume of 200 mL. After overnight culture, PGE2 concentration in the supernatant was determined by ELISA.

### 2.6. Western Blotting

Cell lysis was performed with RIPA buffer (P0013D; Beyotime, Shanghai, China) supplemented with protease (Selleck, Shanghai, China) and phosphatase inhibitors (Selleck). The BCA Protein Assay Kit (PC0020; Solarbio, Beijing, China) was used to measure protein concentrations. The samples were boiled at 100 °C and centrifuged to obtain the supernatant, after which 100 µg of protein was loaded onto 10% sodium dodecyl sulphate-polyacrylamide gel electrophoresis gels (EpiZyme, Shanghai, China). The proteins were transferred to 0.45 µm polyvinylidene fluoride (PVDF) membranes (Millipore, Burlington, MA, USA), and the membranes were blocked in a Western blocking buffer (P0023B; Beyotime) for 2 h at 15 °C. The sections were then incubated with primary antibodies (1:2000) overnight at 4 °C and with the relevant secondary antibody (1:10,000) for 2 h at 15 °C. Bands were visualised using BeyoECL Plus (P0018S; Beyotime). Protein bands were quantified relative to the loading control (GAPDH). The following antibodies were used for Western blotting: anti-GAPDH (AF1186; Beyotime), anti-rabbit-IgG-HRP (A120-111P; Bethyl, Montgomery, TX, USA), and anti-COX2 (ab62331; Abcam, Cambridge, UK).

### 2.7. Statistical Analysis

Statistical analyses were performed using GraphPad Prism version 6.0 (GraphPad Software, San Diego, CA, USA). Statistical differences were determined using unpaired two-tailed t-tests, one-way ANOVA, or two-way ANOVA. The results are shown as the mean ± SEM, and the significance was set at *p* < 0.05. Survival analysis was based on the following criteria: tumour volume, tumour necrosis, and pathological death. Survival analysis was performed using the log-rank test.

## 3. Results

### 3.1. Development of a B16F10 Tumour Cell Line Resistant to Anti-PD-1 Therapy In Vivo

**Figure 1:**
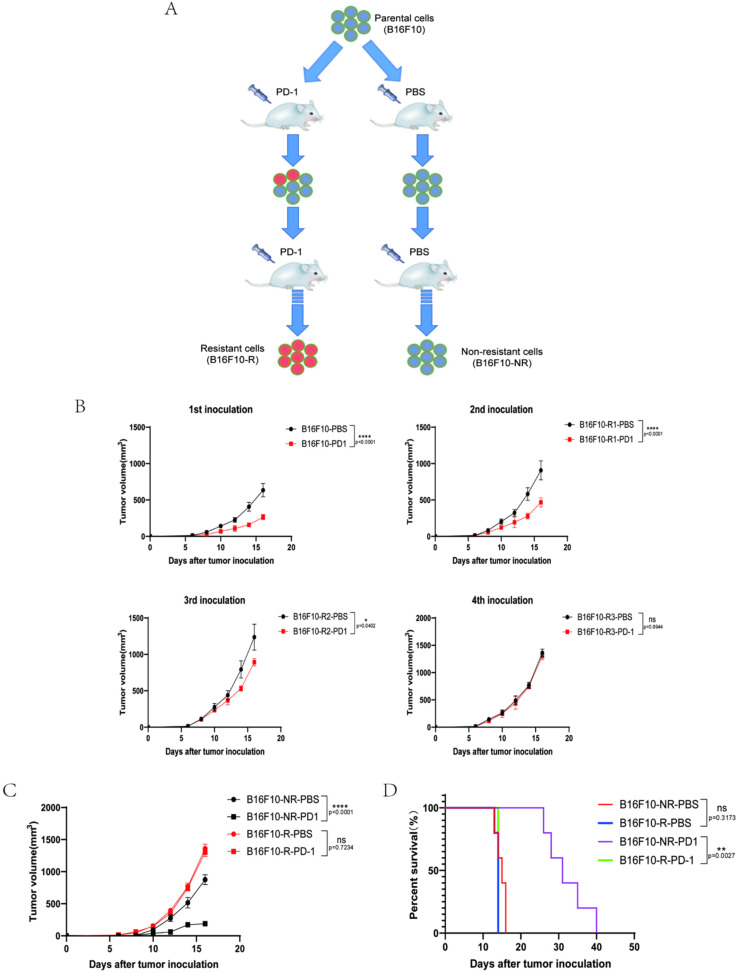
Construction of B16F10 tumour model resistant to anti-PD-1 therapy. (**A**) Construction method of anti-PD-1-resistant B16F10 tumour cells. (**B**) Tumour growth curves for four rounds of anti-PD-1-resistant B16F10 tumour selection (*n* = 6/group, two-way ANOVA test, Sidak). (**C**) Growth curves of B16F10-NR and B16F10-R tumours. Mice were treated with either pembrolizumab or PBS (*n* = 6–8/group, two-way ANOVA test, Tukey). (**D**) Mouse survival curves for B16F10-NR and B16F10-R tumours. Mice were treated with either pembrolizumab or PBS (*n* = 6–8/group, log-rank test of survival curve); ns, not statistically significant; * *p* < 0.05; ** *p* < 0.01; **** *p* < 0.0001.

To acquire a cell line resistant to anti-PD-1 (pembrolizumab) treatment, we established an in vivo B16F10 tumour model using human *Pdcd1* transgenic mice to acquire resistance to anti-PD-1 treatment. In the human *Pdcd1* transgenic mice, the B16F10 tumour cell was sensitive to pembrolizumab treatment. We treated mice with 10 mg/kg pembrolizumab and obtained an anti-PD-1-resistant cell line (B16F10-R) after four rounds of in vivo selection (Figure 1A). Analogously, an anti-PD-1 non-resistant cell line (B16F10-NR) was acquired through four rounds of B16F10 tumour growth in vivo but treated with PBS (Figure 1A). With an increase in the number of rounds of selection, the tumour sensitivity to anti-PD-1 treatment decreased, and there was almost no difference in the fourth round (Figure 1B). Moreover, the resistance persisted through rounds five and six (Figure S1).

This B16F10-NR cell line was very sensitive to anti-PD-1 treatment, but anti-PD-1 therapy did not effectively control B16F10-R tumour growth after four selection rounds (Figure 1C). In addition, anti-PD-1 therapy effectively prolonged the mean survival time of mice in the B16F10-PD-1 group by an average of approximately 3–4 weeks compared with that in the other groups (Figure 1D). 

Thus, the B16F10-R tumour model showed acquired resistance to anti-PD-1 treatment. The persistence of this phenotype in B16F10-R tumour cells after several consecutive in vitro cell cultures suggested that the acquired resistance was caused by genetic changes in the tumour cells. 

### 3.2. The Infiltrating Immune Cells Decreased Significantly in the TME of B16F10-R Tumours

**Figure 2:**
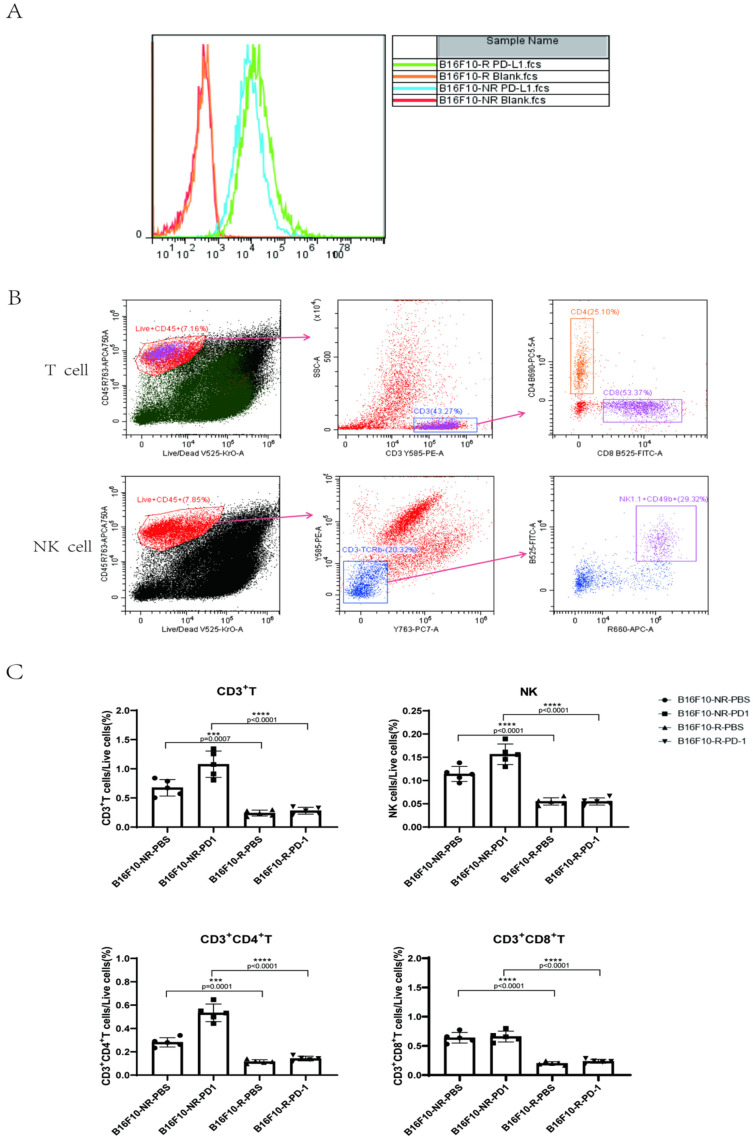
Differences between B16F10-NR tumour and B16F10-R tumour cells. (**A**) PD-L1 expression on the surface of B16F10-NR and B16F10-R cells. (**B**) Gating strategy to identify intratumoural T and NK cells. (**C**) Differences in the number of infiltrating lymphocytes in the TME of B16F10-NR tumours and B16F10-R tumours (one-way ANOVA test, Tukey); ns, not statistically significant; *** *p* < 0.001; **** *p* < 0.0001.

To understand the difference between drug-resistant and non-drug-resistant tumours, we hypothesised that there would be an alteration in PD-L1 expression on the tumour surface. Flow cytometry analysis showed that PD-L1 expression on the surface of B16F10-R tumour cells was slightly increased (Figure 2A). This phenomenon indicated that the tumour cells that acquired anti-PD-1 resistance were not a result of a decrease in the expression of PD-L1. Next, we determined the number of infiltrating lymphocytes in TME. A flow cytometry staining method was developed to distinguish T cells from natural killer (NK) cells (Figure 2B). We observed that infiltration of CD3+, CD4+, and CD8+ T cells was considerably lower in B16F10-R tumours than in B16F10-NR tumours (Figure 2C). In addition, NK cell infiltration significantly decreased (Figure 2C). Thus, we speculated that the acquired resistance of B16F10-R tumours was caused by the decreased infiltration of immune cells.

### 3.3. Aspirin Could Inhibit B16F10-R Tumour Growth

**Figure 3:**
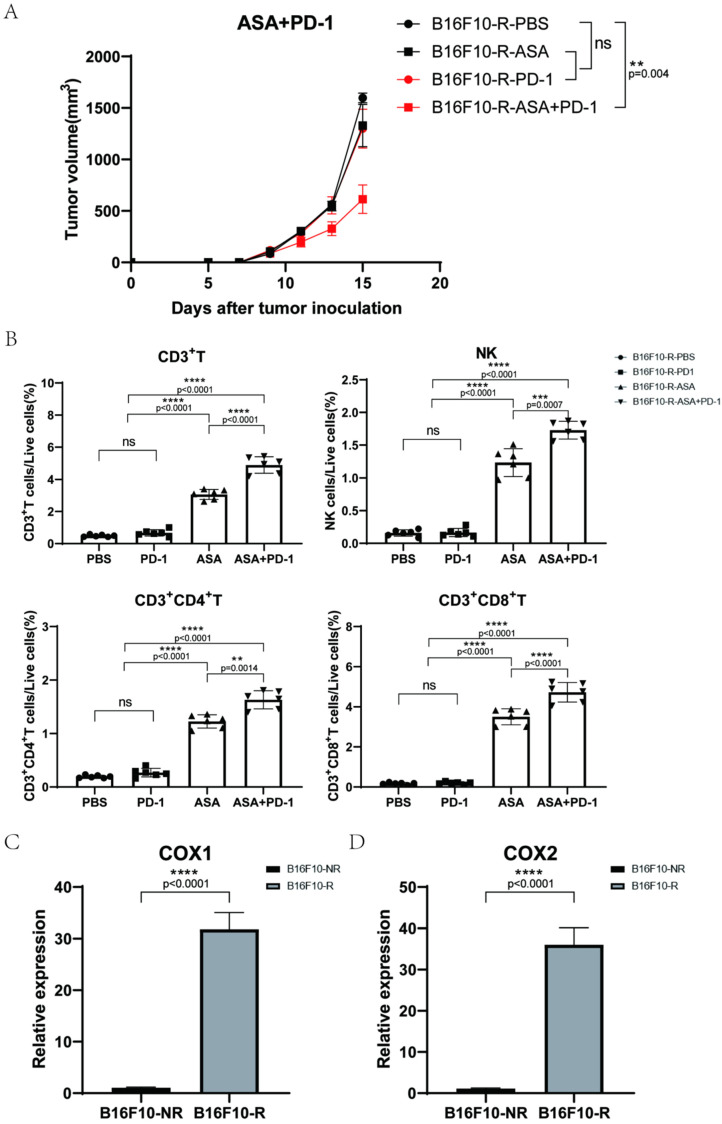
Effects of aspirin combined with pembrolizumab on B16F10-R tumour growth in vivo. (**A**) Growth curves of B16F10-R tumours in vivo treated with pembrolizumab, ASA, or PBS (*n* = 6–8/group, two-way ANOVA test, Tukey). (**B**) Differences in the number of infiltrating lymphocytes in B16F10-R tumours treated with ASA (one-way ANOVA test, Tukey). (**C**,**D**) RT-PCR analysis of *ptgs1* (**C**) and *ptgs2* (**D**) mRNA expression in B16F10-NR and B16F10-R tumours. GAPDH mRNA expression was used as the control (*n* = 8/group, unpaired, two-tailed *t* test); ns, not statistically significant; ** *p* < 0.01; *** *p* < 0.001; **** *p* < 0.0001.

To further confirm whether tumour-acquired resistance to anti-PD-1 therapy was due to decreased immune cell infiltration, we investigated the effects of inhibitors that increased immune cell infiltration. Aspirin is a non-selective inhibitor of COX1/COX2 which can inhibit the expression of COX in tumour cells and then inhibit the production of PGE2, decreasing the infiltration of immune cells into tumours [27]. Next, we evaluated the tumour growth by combination therapy with aspirin and pembrolizumab in B16F10-R tumour cells. Human *Pdcd1* transgenic C57BL/6J mice were injected with aspirin, pembrolizumab, or PBS. Notably, tumour growth in the group treated with pembrolizumab alone was not affected compared to that in the PBS group. In addition, aspirin alone did not inhibit tumour growth. However, the combination of aspirin and pembrolizumab considerably inhibited tumour growth in B16F10-R tumour cells compared with that in the PBS group (Figure 3A).

Next, we examined the TME using flow cytometry analysis, which showed that the infiltration of CD3+, CD4+, and CD8+ T cells was markedly increased in the two aspirin groups with or without pembrolizumab (Figure 3B and Figure S2). In addition, NK cell infiltration notably increased (Figure 3B and Figure S2). These results suggest that aspirin can increase immune cell infiltration in tumours and overcome anti-PD-1 resistance.

### 3.4. COX2 Inhibitor Can Inhibit B16F10-R Tumour Growth and Recover Immune Cell Infiltration

Since aspirin is a non-selective inhibitor that inhibits both COX1 and COX2, we wanted to determine which, if any, played a more important role in tumour-acquired resistance. Thus, we verified the difference in the expression of COX1/COX2 in B16F10-R and B16F10-NR tumour tissues via RT-qPCR to estimate the relative abundance of COX1/COX2 mRNA. B16F10-R tumours exhibited significantly higher mRNA levels of both COX1 and COX2 than B16F10-NR tumours (Figure 3C,D).

**Figure 4:**
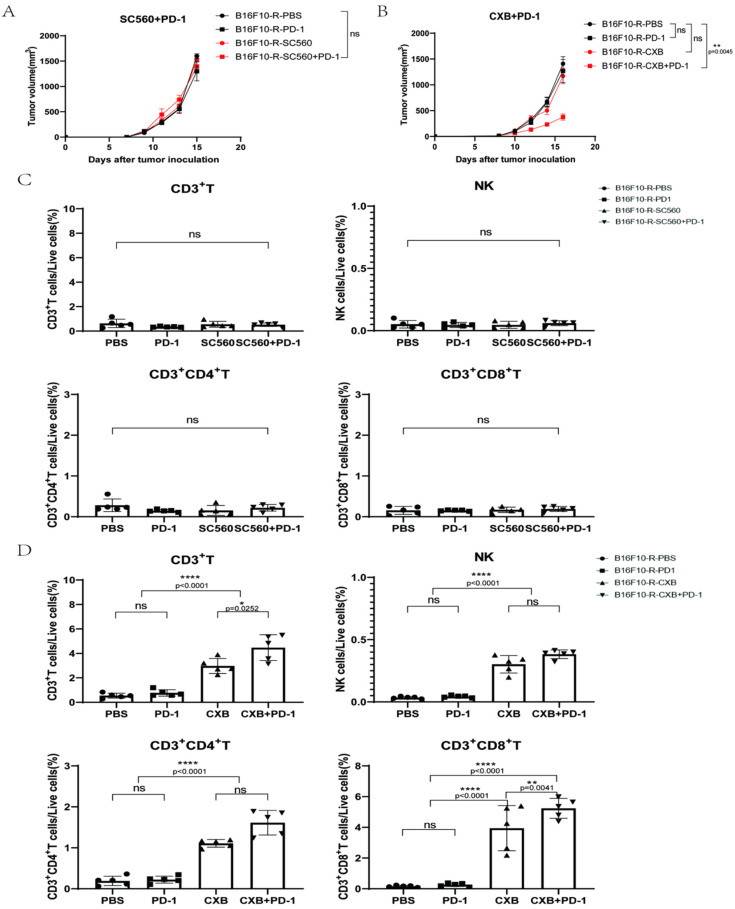
Effects of SC560 or celecoxib combined with pembrolizumab on B16F10-R tumour growth in vivo. (**A**) Growth curves of B16F10-R tumours in vivo treated with pembrolizumab, SC560, or PBS (*n* = 6–8/group, two-way ANOVA test, Tukey). (**B**) Growth curves of B16F10-R tumours in vivo treated with pembrolizumab, celecoxib, or PBS (*n* = 6–8/group, two-way ANOVA test, Tukey). (**C**) Differences in the number of infiltrating lymphocytes cells in B16F10-R tumours treated with celecoxib (one-way ANOVA test, Tukey). (**D**) Differences in the number of infiltrating lymphocytes in B16F10-R tumours treated with SC560 (one-way ANOVA test, Tukey); ns, not statistically significant; * *p* < 0.05; ** *p* < 0.01; **** *p* < 0.0001.

To determine whether COX1 or COX2 was more vital, the selective COX1 inhibitor SC560 and selective COX2 inhibitor celecoxib were used in combination with pembrolizumab. We found that SC560 combined with pembrolizumab did not affect tumour growth (Figure 4A), whereas celecoxib effectively restricted tumour growth when combined with pembrolizumab (Figure 4B). In addition, nimesulide, another selective COX2 inhibitor, inhibited tumour growth in combination with pembrolizumab (Figure S3). Moreover, lymphocyte infiltration in the TME increased significantly after celecoxib treatment (Figure 4D and Figure S4). However, these phenomena were not detected in the SC560 group (Figure 4C and Figure S5). In addition, we also found that B16F10-R tumours exhibited significantly higher protein levels of COX2 than B16F10-NR tumours (Figure S6). Consistently, we also found that B16F10-R tumours secreted significantly higher levels of PGE2 than B16F10-NR tumours in vitro (Figure S7A). These results indicated that COX1 had no effect, but COX2 had a crucial effect on the development of acquired resistance in tumours. Thus, the inhibition of COX2 could effectively overcome drug resistance.

### 3.5. COX2 Knockout Abrogated the Acquired Resistance to Anti-PD-1 Treatment in B16F10-R Tumour Cells

**Figure 5:**
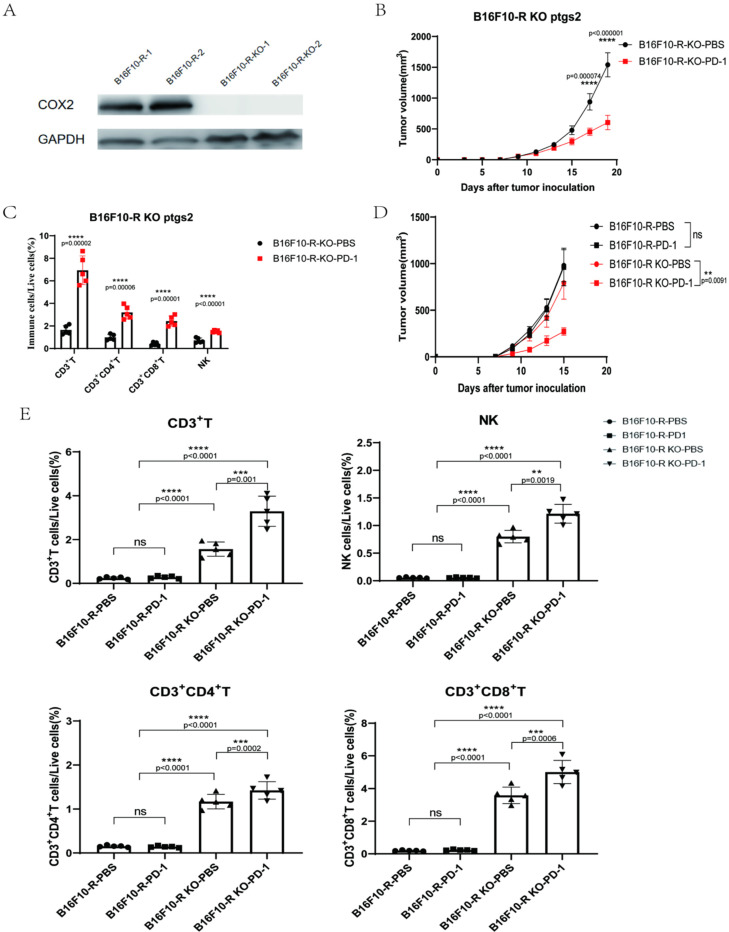
COX2 knockout in B16F10-R cells abrogates acquired resistance to anti-PD-1 therapy. (**A**) Western blot analysis for COX2 expression. GAPDH was the control in the two cell lines. (**B**) Tumour growth curves of B16F10-R-knockout tumours in vivo treated with pembrolizumab or PBS (*n* = 6–8/group, two-way ANOVA test, Tukey). (**C**) Differences in the number of infiltrating lymphocytes in B16F10-R-knockout tumours (one-way ANOVA test, Tukey). (**D**) Tumour growth curves of B16F10-R and B16F10-R-knockout tumours in vivo treated with pembrolizumab or PBS (*n* = 6–8/group, two-way ANOVA test, Tukey). (**E**) Differences in the number of infiltrating lymphocytes in B16F10-R-knockout tumours (one-way ANOVA test, Tukey); ns, not statistically significant; ** *p* < 0.01; *** *p* < 0.001; **** *p* < 0.0001.

To further verify the role of COX2 in the development of acquired resistance to anti-PD-1 treatment, we knocked out COX2 in B16F10-R tumour cells (Figure 5A and Figure S6), and we found that PGE2 secretion was significantly reduced in B16F10-R-knockout tumours compared with B16F10-R tumours in vitro (Figure S7B). After COX2 knockout, anti-PD-1 therapy exhibited obvious therapeutic effects on drug-resistant tumours (Figure 5B), where lymphocyte infiltration was significantly increased in the TME (Figure 5C). Next, we compared the growth of B16F10-R tumours and B16F10-R-knockout tumours following anti-PD-1 treatment. There was no significant change in tumour growth without anti-PD-1 treatment; however, tumour growth was significantly inhibited after anti-PD-1 treatment (Figure 5D). Flow cytometry analysis showed that B16F10-R-knockout tumours had significantly more infiltrating immune cells than B16F10-R tumours did (Figure 5E and Figure S8). This indicated that the increased infiltration of lymphocytes was caused by the knockout of COX2. However, without pembrolizumab, the tumour would have immune escape due to the PD-1/PD-L1 pathway. Moreover, the continuous blocking of the PD-1/PD-L1 pathway can have significant inhibitory effects.

### 3.6. EP4 Inhibitor Could Inhibit B16F10-R Tumour Growth

**Figure 6:**
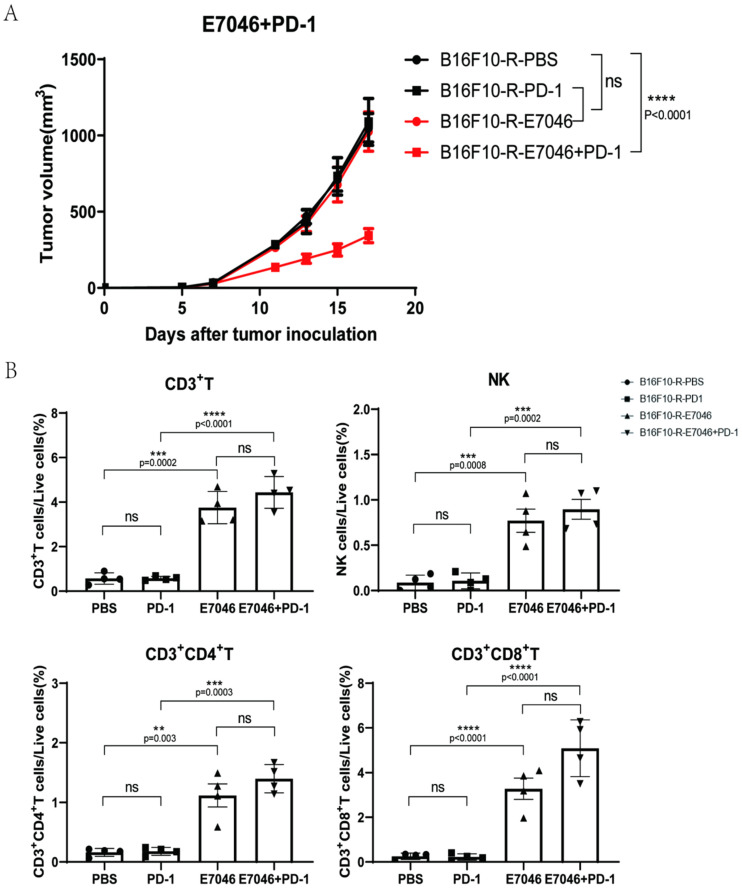
Effects of E7046 combined with pembrolizumab on B16F10-R tumour growth in vivo. (**A**) Growth curves of B16F10-R tumours in vivo treated with pembrolizumab, E7046, or PBS (*n* = 6–8/group, two-way ANOVA test, Tukey). (**B**) Differences in the number of infiltrating lymphocytes in B16F10-R tumours treated with E7046 (one-way ANOVA test, Tukey); ns, not statistically significant; ** *p* < 0.01; *** *p* < 0.001; **** *p* < 0.0001.

Most of the functions of PGE2 are mediated by four PGE2 receptors, EP1, EP2, EP3, and EP4 [28]. PGE2 inhibits the killing, cytokine production, and chemotactic activity of tumour target cells by interacting with EP4, which is expressed on NK cells [29]. EP4 is associated with drug resistance in tumours [30,31]. Therefore, we hypothesised that EP4 is related to acquired resistance to anti-PD-1 therapy in B16F10-R tumours. We used E7046, a selective EP4 inhibitor, in combination with pembrolizumab to treat B16F10-R tumours in vivo. We found that E7046 combined with pembrolizumab inhibited tumour growth; however, the other two groups showed no significant inhibition compared to the PBS group (Figure 6A). Additionally, infiltrating immune cells were detected in the TME. We observed that the infiltrating immune cells contained CD3+, CD4+, and CD8+ T cells, as well as NK cells, and that their numbers increased significantly in the E7046 and E7046 + PD-1 groups relative to the other two groups (Figure 6B and Figure S9). These results indicated that the EP4 receptor may be associated with the acquisition of resistance to anti-PD-1 therapy in B16F10-R cells. Thus, the inhibition of EP4 receptors can effectively address drug resistance.

## 4. Discussion

Malignant tumours can lead to the inactivation of cytotoxic T cells after PD-1 binds to its ligand via the upregulation of PD-L1 expression. Therefore, anti-PD1 or anti-PD-L1 monoclonal antibodies can restore the immune inhibition of tumour growth by blocking the PD-1/PD-L1 pathway. In several cancer types, pembrolizumab has considerably increased patient survival through its therapeutic inhibition of PD-1 [32]. Although anti-PD-1 or anti-PD-L1 drugs are promising for cancer therapy, there are still many problems to be solved. In general, the objective response rates of anti-PD-1 or anti-PD-L1 monoclonal antibodies are nearly 10–50% in most patients [33]. In addition, patients treated with pembrolizumab for a long time are prone to drug resistance [34], thus allowing the tumour to use other signalling pathways for immune escape. Therefore, it is important to study how tumour cells develop resistance to anti-PD-1 treatment.

In this study, we established a melanoma mouse model of B16F10 tumour resistance to anti-PD-1 treatment using four rounds of pembrolizumab therapy and selection in vivo. Additionally, human Pdcd1 transgenic C57BL/6J mice were used to study secondary drug resistance. After four rounds of treatment, B16F10 tumours became resistant to pembrolizumab in vivo. This resistance persisted in cultured B16F10 cells in vitro, suggesting that the acquired resistance was caused by hereditary changes in tumour cells.

The detection of PD-L1 expression on the tumour surface ruled out the possibility that a decrease in its expression led to immune escape of the B16F10-R tumour. Flow cytometry analysis of the TME revealed that the number of infiltrating immune cells, including CD3+, CD4+, and CD8+ T cells, as well as NK cells, was considerably reduced in B16F10-R tumours compared to that in B16F10-NR tumours. We speculated whether the reduction in immune cell infiltration in B16F10-R tumours resulted in tumour immune escape against anti-PD-1 therapy.

The COX2-PGE2 pathway has been reported to be involved in the infiltration of NK and T cells in tumours [30]. In addition, it has also been reported that COX inhibitors can treat tumour patients who have been resistant to other therapies [35]. Thus, inhibiting COX2 may provide a chance to reduce immune escape in tumours [22,23,24,25,26,27,28,29,30,31,32,33,34,35,36,37]. Therefore, we used aspirin to treat drug-resistant tumours and showed that aspirin alone did not affect tumour growth, but its combination with pembrolizumab significantly inhibited tumour growth in mice.

RT-qPCR results showed that the expression of COX1/COX2 was significantly higher in drug-resistant tumours than in wild-type tumours. The combination of selective inhibitors of COX1/COX2 with pembrolizumab indicated that COX2 may be the main reason for the development of drug resistance. Therefore, we knocked out the *ptgs2* gene in drug-resistant tumour cells to verify the function of COX2 in tumour drug resistance. After knockout, the sensitivity of drug-resistant tumours to pembrolizumab changed significantly. In addition, infiltrating lymphocytes in the TME increased dozens of times. These results illustrate that COX2 expression is highly correlated with tumour drug resistance.

Subsequently, we targeted EP4 receptors, which were one of downstream receptors of PGE2, and found that EP4 was associated with drug resistance in some tumours. Moreover, we found that E7046, an inhibitor of EP4, combined with pembrolizumab, inhibited the growth of drug-resistant tumours and restored lymphocyte infiltration in the TME. This result also revealed the crucial function of the COX2/PGE2/EP4 pathway in tumour drug resistance.

In all the animal experiments related to drug-resistant tumours, we observed two common phenomena. First, the use of a single inhibitor or pembrolizumab alone did not affect tumour growth, and only a combination of therapies inhibited tumour growth. Second, as long as COX2-related inhibitors were used, the number of infiltrating immune cells in the tumours increased significantly. To explain these two phenomena, we postulated that after the tumour acquired drug resistance, the expression of PD-L1 on the tumour cell surface did not decrease, but the lymphocytes in the tumour were significantly reduced. When only pembrolizumab was used, no effect was observed, probably due to the extreme lack of immune cells. When only COX2-related inhibitors were used, the lymphocytes in the tumour recovered, but the tumour still expressed PD-L1, and immune escape was carried out through the PD-1/PD-L1 pathway; hence, there was no overall effect. Tumour growth can only be effectively inhibited by simultaneous blocking of the PD-1/PD-L1 pathway and restoration of lymphocyte infiltration in the TME with COX2 inhibitors.

In summary, upregulation of COX2 might be the reason for tumour-acquired resistance to anti-PD-1 therapy. Our work demonstrated that COX2 inhibitors could promote pembrolizumab efficacy and inhibit tumour growth in a drug-resistant model. Mechanistically, celecoxib inhibited the COX2 effect and suppressed PGE2 production, which in turn elevated the infiltration of CD3+, CD4+, and CD8+ T cells, as well as NK cells, resulting in the suppression of tumour growth. 

Tumour drug resistance is a complex process caused by a range of situations. The results of our study may only reveal one of the causes of tumour-acquired resistance to anti-PD-1 therapy, and further research, including clinical tests, is needed to verify their applicability. Nonetheless, our findings further our understanding of the mechanisms underlying drug resistance in tumours. Furthermore, we identified a new role for the COX2 signalling pathway in anti-PD-1-resistant tumours. Our study suggests that elevated COX2 expression is a potential biomarker of poor immunotherapy response in anti-PD-1-resistant tumours. The dual targeting of PD-1 and COX2 in tumours may enhance the efficacy of immune checkpoint therapy and overcome drug resistance to anti-PD-1 therapy. This is an important addition to our current understanding of tumour-acquired resistance to anti-PD-1 therapy and provides research directions for developing a clinical treatment for anti-PD-1-resistant tumours and immunotherapy.

## 5. Conclusions

In conclusion, we developed an anti-PD-1-resistant B16F10 tumour model using human Pdcd1 transgenic mice. Lymphocyte infiltration was significantly reduced and COX2 gene expression was increased in B16F10-R tumours compared with B16F10-NR tumours. Moreover, COX2 inhibitors could restore the immune cell infiltration in tumour, and the combination with pembrolizumab could inhibit tumour growth again, lifting the limitation of drug resistance. Therefore, the combination of COX2 inhibitors and anti-PD-1 mAbs may become a new choice for the drug resistance of anti-PD-1 therapy in the future.

## Data Availability

All data relevant to the study were included in the article or uploaded as supplemental online information.

## Supplementary Materials

The following supporting information can be downloaded at: https://www.mdpi.com/article/10.3390/cancers14174134/s1, Figure S1: Construction of B16F10 tumour model resistant to anti-PD-1 therapy. (A,B) Tumour growth curves for rounds five and rounds six of anti-PD-1 resistant B16F10 tumour selection (n = 6/group, two-way ANOVA test, Sidak); Figure S2: Flow scatter diagrams of lymphocyte infiltration in B16F10-R tumours treated with ASA. (A) Flow scatter diagram of T cell infiltration. (B) Flow scatter diagram of NK cell infiltration; Figure S3: Tumour growth curves of B16F10-R tumour in vivo treated with pembrolizumab, nimesulide or PBS (n = 6–8/group, two-way ANOVA test, Tukey); Figure S4: Flow scatter diagrams of lymphocyte infiltration in B16F10-R tumours treated with CXB. (A) Flow scatter diagram of T cell infiltration. (B) Flow scatter diagram of NK cell infiltration; Figure S5: Flow scatter diagrams of lymphocyte infiltration in B16F10-R tumours treated with SC560. (A) Flow scatter diagram of T cell infiltration. (B) Flow scatter diagram of NK cell infiltration; Figure S6: Western blot analysis for COX2 expression. GAPDH was the control in the two cell lines(one-way ANOVA test, Tukey); Figure S7: Concentration of PGE2 in the supernatant of tumour cells cultured in vitro. (A) Concentration of PGE2 of B16F10-NR or B16F10-R tumour cells cultured in vitro which was determined by ELISA (n = 6/group, unpaired, two-tailed *t* test). (B) Concentration of PGE2 of B16F10-R or B16F10-R-knockout tumour cells cultured in vitro which was determined by ELISA (n = 6/group, unpaired, two-tailed *t* test); Figure S8: Flow scatter diagrams of lymphocyte infiltration in B16F10-R or B16F10-R-knockout tumours. (A) Flow scatter diagram of T cell infiltration. (B) Flow scatter diagram of NK cell infiltration; Figure S9: Flow scatter diagrams of lymphocyte infiltration in B16F10-R tumours treated with E7046. (A) Flow scatter diagram of T cell infiltration. (B) Flow scatter diagram of NK cell infiltration.

## Abbreviations

PD-1	programmed cell death protein 1
PD-L1	programmed death-ligand 1
COX2	cyclooxygenase-2
PGE2	prostaglandin E2
TME	tumour microenvironment
NK cells	natural killer cells
