# Supplementary material for: Reversing PD-1 Resistance in B16F10 Cells and Recovering Tumour Immunity Using a COX2 Inhibitor
Source: Cancers (Basel). 2022 Aug 26;14(17):4134. doi: 10.3390/cancers14174134 (PMC9455073; doi:10.3390/cancers14174134)
Supplement: Supplementary file 1 [file cancers-14-04134-s001.zip › cancers-1848175-supplementary.pdf]

Supplementary Materials

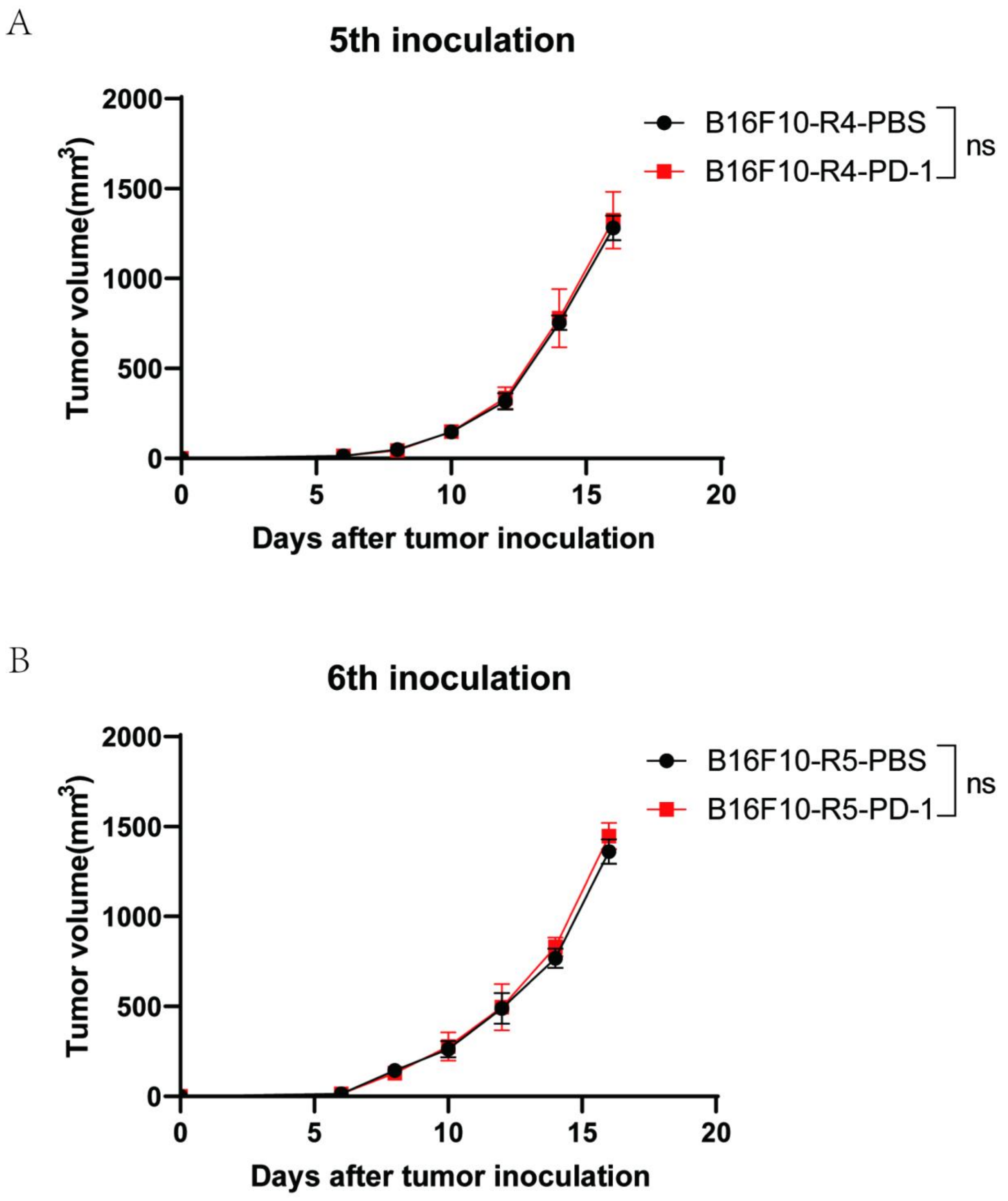

Figure S1. Construction of B16F10 tumour model resistant to anti-PD-1 therapy.

(A-B) Tumour growth curves for rounds five and rounds six of anti-PD-1 resistant B16F10 tumour selection(n = 6/group, two-way ANOVA test, Sidak).

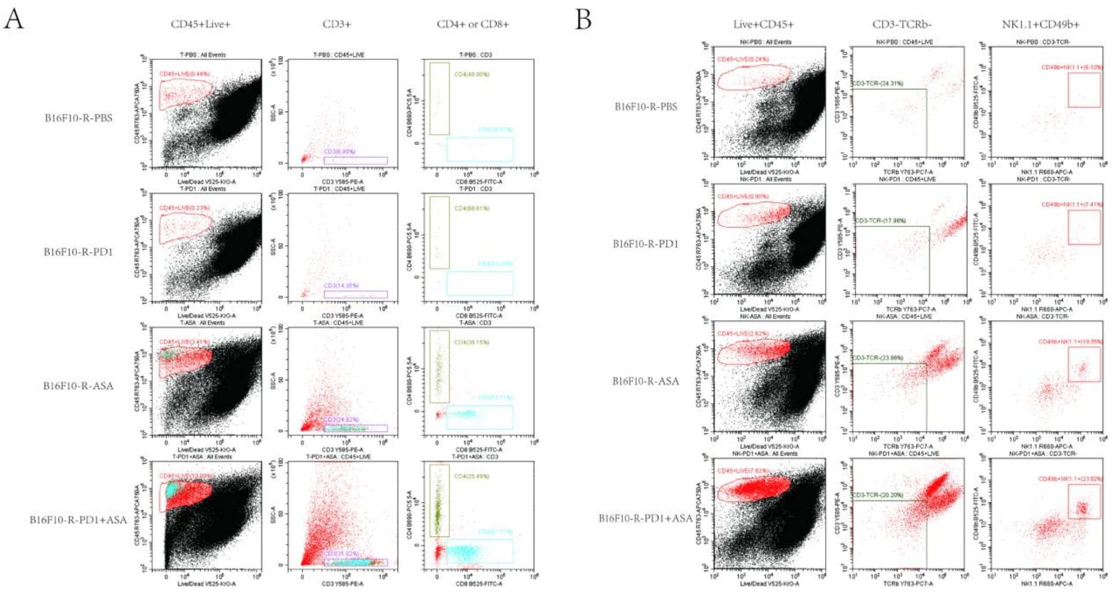

Figure S2. Flow scatter diagrams of lymphocyte infiltration in B16F10-R tumours treated with ASA.

(A) Flow scatter diagram of T cell infiltration. (B) Flow scatter diagram of NK cell infiltration.

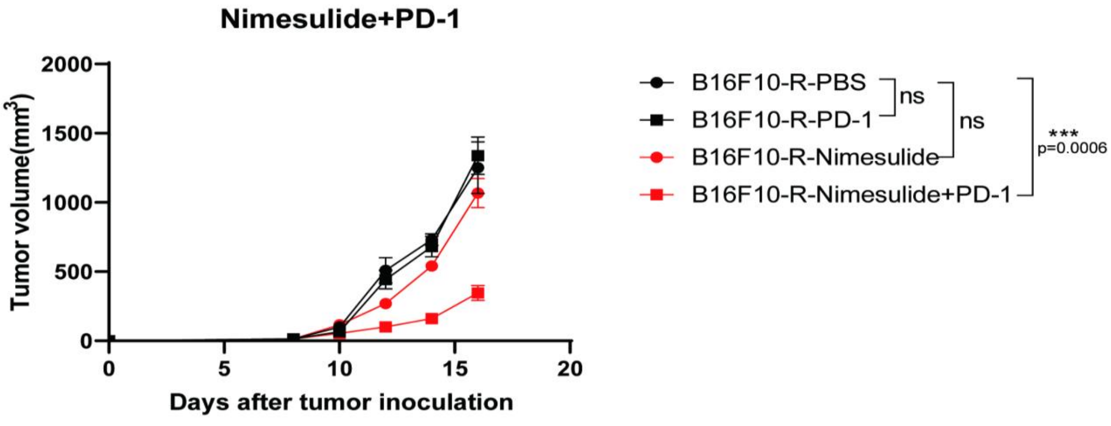

Figure S3. Tumour growth curves of B16F10-R tumour in vivo treated with pembrolizumab, nimesulide or PBS (n = 6–8/group, two-way ANOVA test, Tukey).

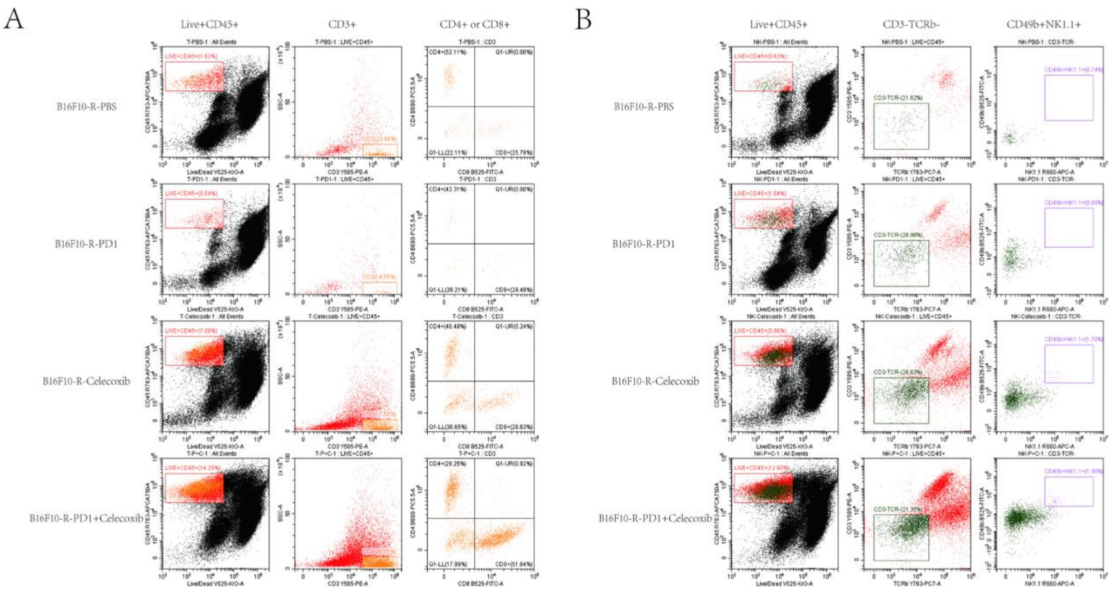

Figure S4. Flow scatter diagrams of lymphocyte infiltration in B16F10-R tumours treated with CXB.

(A) Flow scatter diagram of T cell infiltration. (B) Flow scatter diagram of NK cell infiltration.

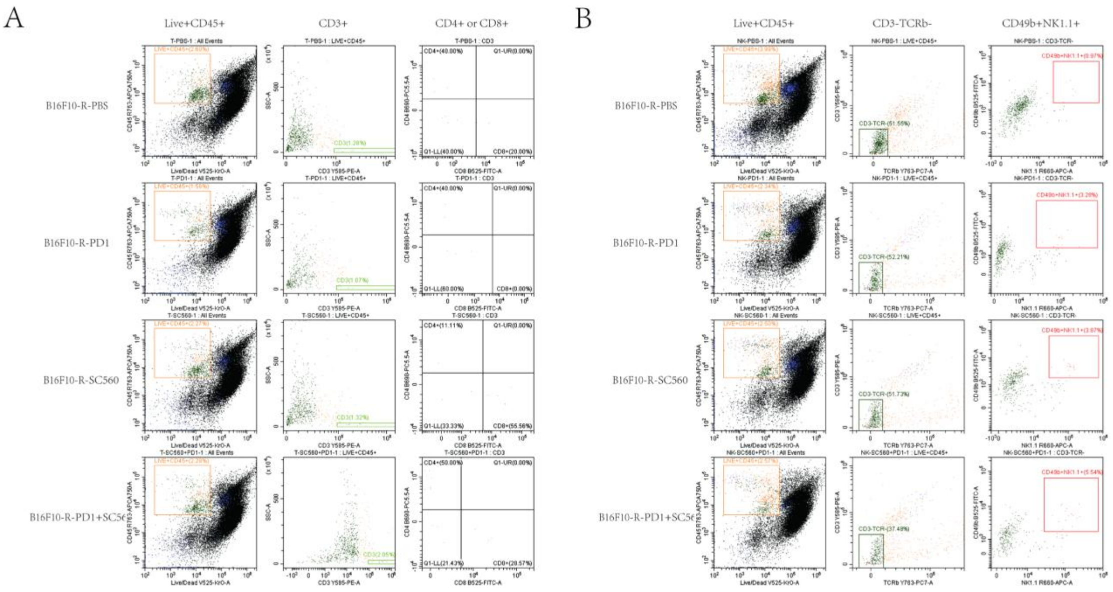

Figure S5. Flow scatter diagrams of lymphocyte infiltration in B16F10-R tumours treated with SC560.

(A) Flow scatter diagram of T cell infiltration. (B) Flow scatter diagram of NK cell infiltration.

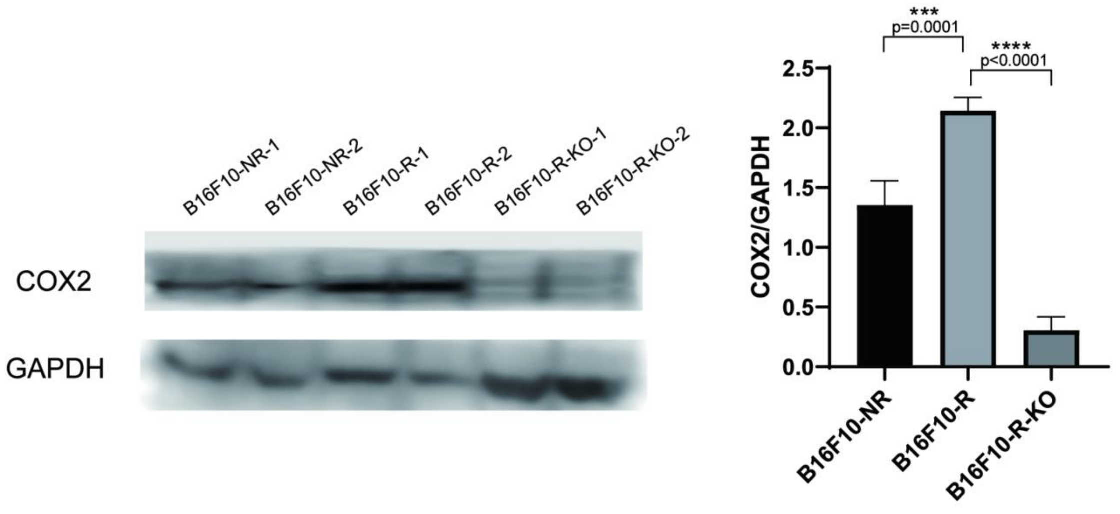

Figure S6. Western blot analysis for COX2 expression. GAPDH was the control in the two cell lines(one-way ANOVA test, Tukey).

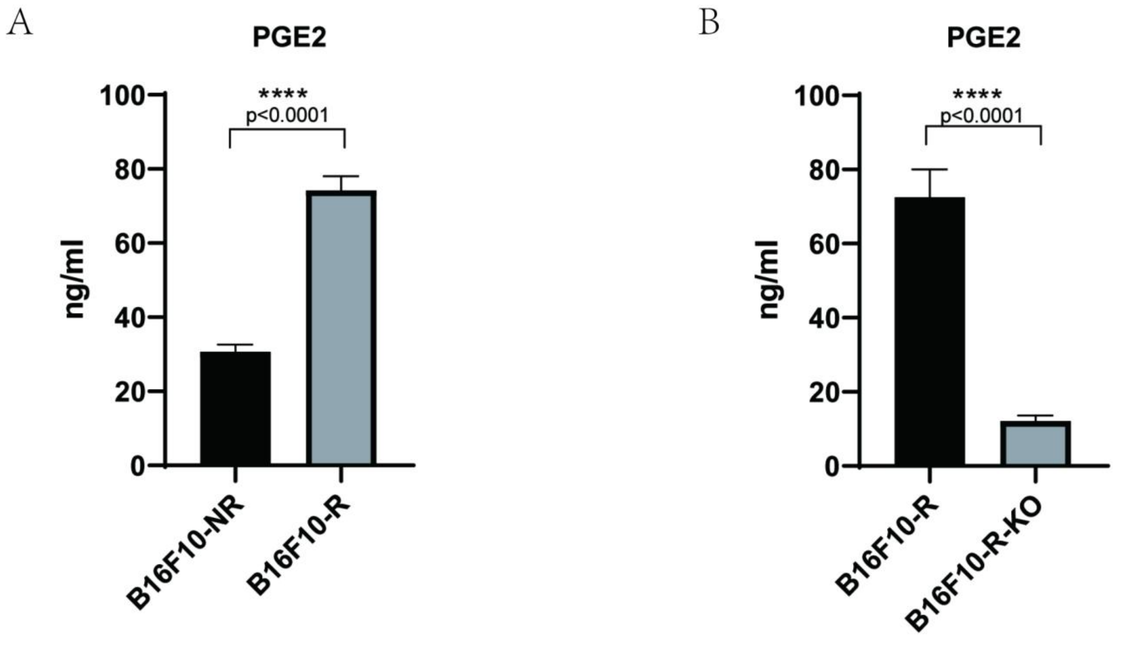

Figure S7. Concentration of PGE2 in the supernatant of tumour cells cultured in vitro.

(A) Concentration of PGE2 of B16F10-NR or B16F10-R tumour cells cultured in vitro which was determined by ELISA(n = 6/group, unpaired, two-tailed t test). (B) Concentration of PGE2 of B16F10-R or B16F10-R-knockout tumour cells cultured in vitro which was determined by ELISA(n = 6/group, unpaired, two-tailed t test).

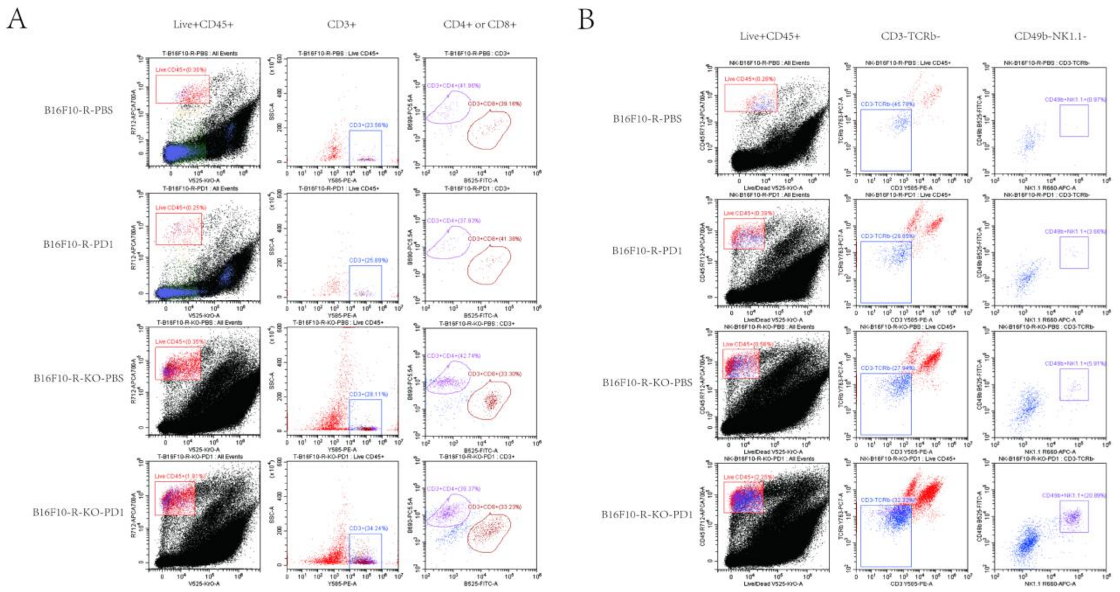

Figure S8. Flow scatter diagrams of lymphocyte infiltration in B16F10-R or B16F10-R-knockout tumours.

(A) Flow scatter diagram of T cell infiltration. (B) Flow scatter diagram of NK cell infiltration.

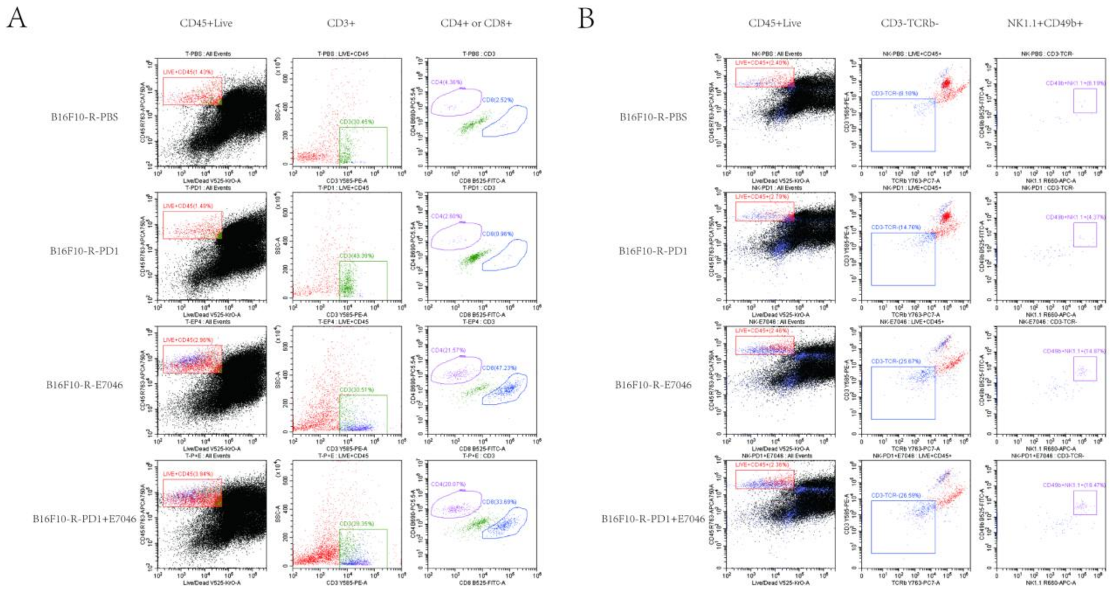

Figure S9. Flow scatter diagrams of lymphocyte infiltration in B16F10-R tumours treated with E7046.

(A) Flow scatter diagram of T cell infiltration. (B) Flow scatter diagram of NK cell infiltration.
